# Supplementary material for: How to build a ribosome from RNA fragments in Chlamydomonas mitochondria
Source: Nat Commun. 2021 Dec 9;12:7176. doi: 10.1038/s41467-021-27200-z (PMC8660880; doi:10.1038/s41467-021-27200-z)
Supplement: Supplementary file 1 — Supplementary Information [file 41467_2021_27200_MOESM1_ESM.pdf]

a

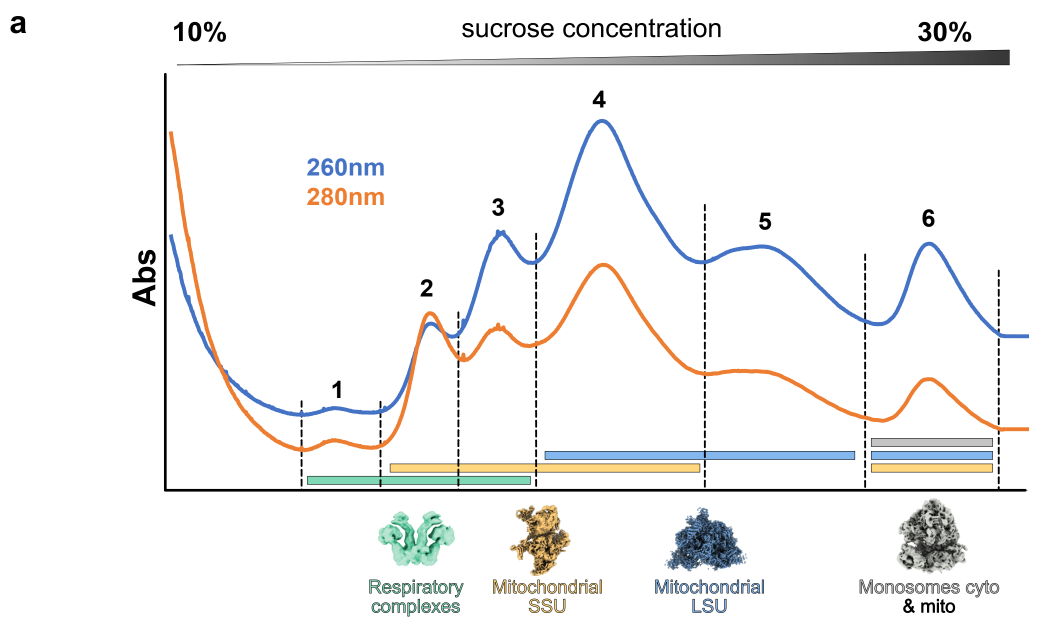

b

Protein	Uniprot ID	Total	Mito	Fractions 1	2	3	4	5	6
**ATP Synthase (subunit 19.5 kDa)**	Q6QAY3	16	95	180	154	106	65	20	13
**ATP Synthase (subunit 5)**	A8J3U9	7	84	165	158	95	50	8	9
**ATP Synthase (subunit 36.3 kDa)**	A8JCE9	23	85	146	169	90	50	20	17
**Complex I (subunit 39 kDa)**	Q6V506	12	77	35	141	66	18	4	0
**Complex I (subunit 24 kDa)**	Q6V9B3	14	36	21	60	35	14	2	0
**Complex I (subunit 49 kDa)**	Q6V9A8	10	66	29	106	61	13	2	0
**bS1m**	A8IB56	0	3	0	15	51	29	14	7
**uS2m**	A0A2K3CXL0	0	5	0	22	83	43	32	16
**uS4m**	A0A2K3E198	0	13	5	37	83	51	50	19
**uS9m**	A0A2K3E2Q3	0	15	0	30	90	45	41	10
**bTHXm**	A8J1A6	0	0	0	0	16	11	8	0
**mS26**	A8J4B1	0	4	0	8	30	11	14	6
**uL3m**	A0A2K3E681	0	4	1	8	50	95	58	34
**uL13m**	A8J810	0	1	0	0	11	26	23	5
**uL15m**	A0A2K3D8F3	0	4	0	3	23	47	40	12
**bL33m**	A8J9C3	0	0	0	0	5	11	11	4
**mL64/L59**	A8HPK3	0	0	0	0	11	38	35	9
**mL46**	A8IWI1	0	3	0	0	6	43	33	13
**SSU Cyto (uS2)**	A8IB25	22	0	0	0	2	0	0	10
**SSU Cyto (eS6)**	A8J1G8	25	0	0	0	6	2	5	26
**SSU Cyto (uS15)**	A8J493	19	0	3	1	2	3	1	10
**LSU Cyto (uL15)**	A8I2T0	21	0	0	0	0	0	2	13
**LSU Cyto (uL24)**	A8HMG7	26	0	0	0	0	0	5	14
**LSU Cyto (eL28)**	A0A2K3D7C4	14	0	0	0	0	0	0	13

Newly identifie *C. reinhardtii* specifi mitoribosome proteins

Protein	Uniprot ID	Total	Mito	1	2	3	4	5	6
**mS105**	A0A2K3DAY3	1	42	4	20	65	35	22	12
**mS106**	A0A2K3DZV7	0	10	0	1	75	15	6	1
**mS107**	A0A2K3CRX4	1	5	6	43	119	58	50	31
Uncharacterized protein	A8HUB6	0	12	2	21	47	23	22	15
Uncharacterized protein	A0A2K3DVI2	0	26	7	77	190	112	81	28
**mL113**	A0A2K3DVD3	0	15	9	25	93	166	127	65
**mL114**	A0A2K3DR53	0	1	0	4	39	80	49	31
**mL115**	A8J070	0	5	8	0	36	93	39	11
**mL116**	A0A2K3DXQ3	0	13	10	18	79	132	76	38
**mL117**	A0A2K3CXJ4	0	20	6	4	67	112	76	44
**mL118**	A0A2K3DTE5	0	4	4	14	70	133	73	31
**mL119**	A0A2K3D424	0	4	0	2	39	66	63	31
Uncharacterized protein	A0A2K3CVT3	0	0	0	0	7	31	15	4
Uncharacterized protein	A0A2K3DBX4	0	8	0	5	21	62	39	12
AAA domain-containing protein	A0A2K3CZG9	1	32	2	0	3	44	78	18

Less abundant — Most abundant

Supplementary Fig. 1. *C. reinhardtii* mitoribosome purificatio and identificatio of novel ribosomal components

a) Continuous sucrose gradient profil (260 and 280 nm absorbance). 6 peaks were analyzed by proteomics and screened by cryo-EM. Repartition and reconstructions of the complexes identifie are shown below the chromatogram. **b)** Summary of the proteomic analysis of the corresponding fractions presented in **a**, that allowed the identificatio of novel r-proteins. Total cell (Total) and purifie mitochondria (Mito) fraction MS analyses are also shown here. The abundance of the respective proteins is represented as absolute spectra values colored such that higher spectra values are red and lower are blue. The upper part of the table presents known components of the complexes identifie that were used as references to identify novel components of the LSU and SSU of *C. reinhardtii* mitoribosome. The novel components are presented in the lower part of the table. Proteins that were confirme by cryo-EM are shown in bold.

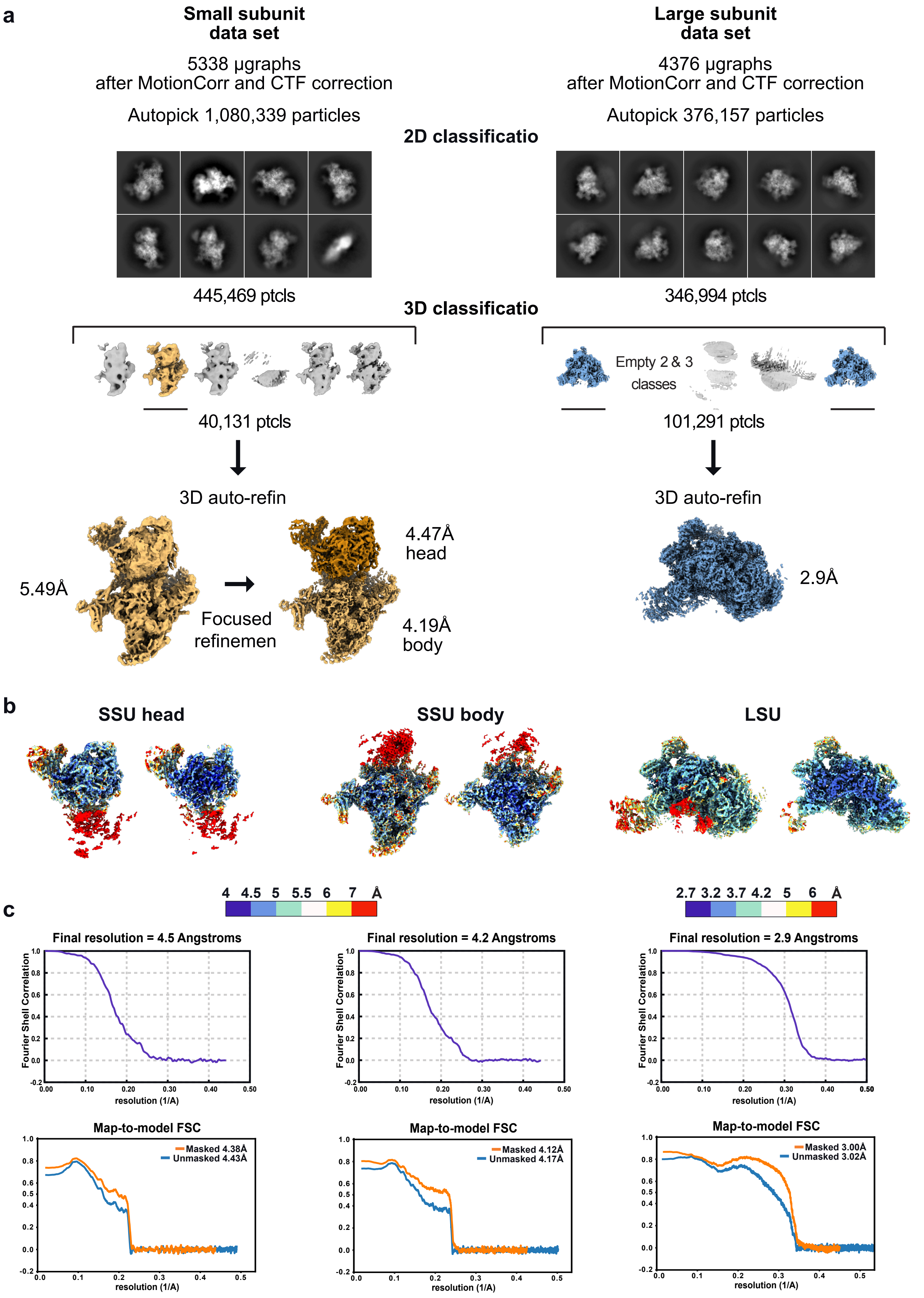

Supplementary Fig. 2. Single-particle data processing workflo

a) Graphical summary of the processing workflo described in Methods, with 2D and 3D classes, processing and refinement **b)** Local resolutions of both the LSU and each focused SSU are presented. The maps are colored by resolution, generated using ResMap[74]. Maps are also shown in cut view. **c)** For each reconstructions FSC plots (output from RELION[75]) are displayed for resolution estimation. Map to model FSC are also shown (output from PHENIX[76] validation), for the SSU, the head part of the SSU model was used against the head map and the body part of the model against the body map. The maps resolution were calculated at the 0.143 threshold.

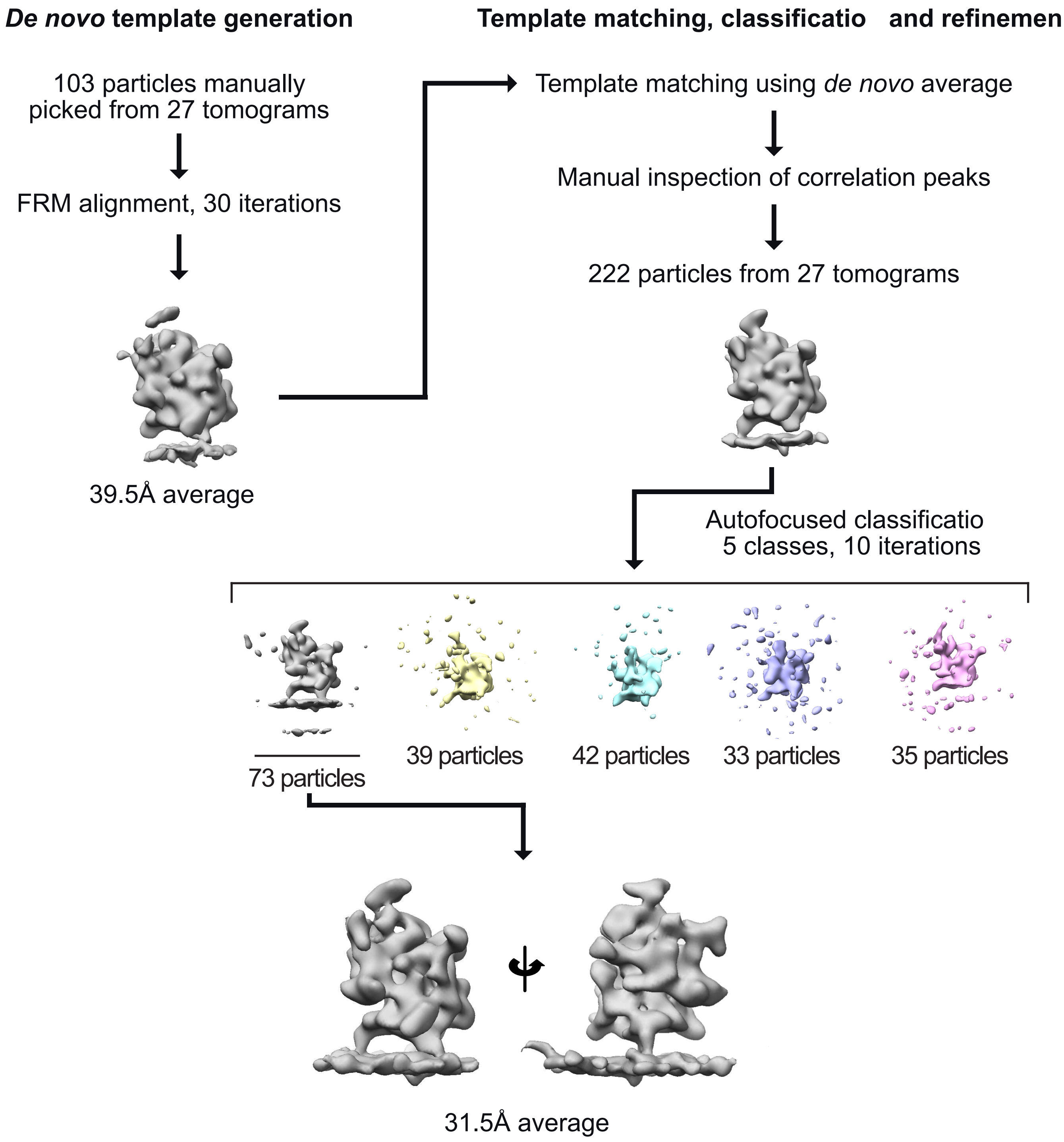

Supplementary Fig. 3. Subtomogram averaging workflow
To create an initial mitoribosome reference, 103 particles were manually picked and subjected to 30 iterations of FRM alignment resulting in an average of 39.5 Å (top left). This was used as a reference volume for Template matching, followed by manual inspection of the top correlation peaks and resulting in a set of 222 particles from 27 tomograms. The 222 particles were aligned and subjected to autofocused classification resulting in one class of 73 particles that showed distinct ribosomal features. 3D refinemen of that class resulted in an average of 31.5 Å. A similar workflo , detailed in the methods section, was adopted for the STA of cytoribosomes and ATP synthases.

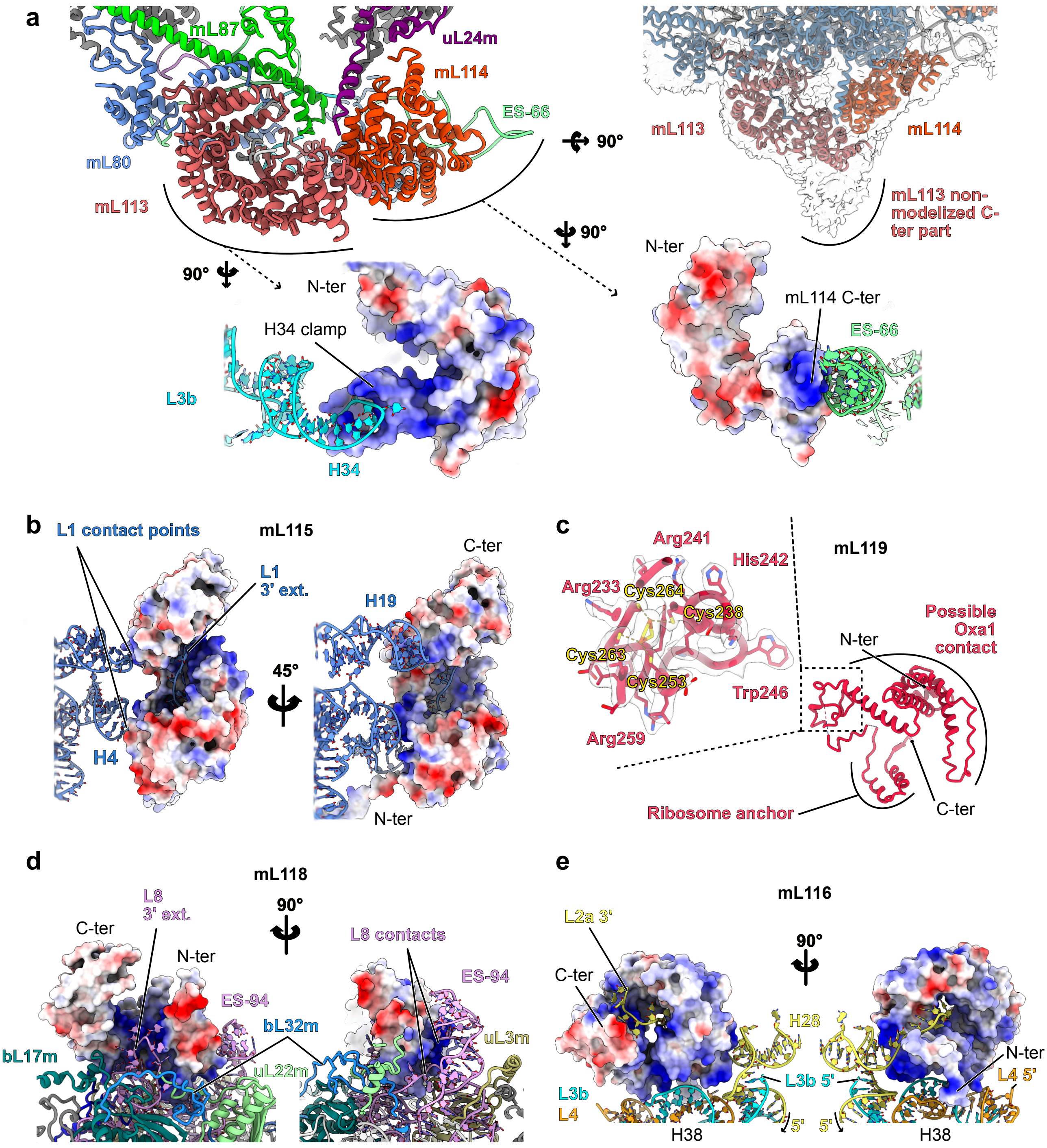

Supplementary Fig. 4. Detailed analyses and views of the specifi LSU proteins

Focused views of the differen Chlamydomonas-specifi r-proteins of the LSU. **a)** View of the mL113 – mL114 area. Both proteins interact together and with the surrounding proteins. The non-modelized C-terminal part of mL113, interacting with the membrane, is shown. It interacts with H34 of the L3b fragment. mL114 interacts with ES-66 of the L7 fragment. Electrostatic coloration (blue coloration corresponds to positive patches and red to negative patches) of the proteins correlate with their rRNA binding site. **b)** Electrostatic coloration of the mL115 protein reveal that its inner groove is mostly positively charged similarly to the two contact points made with H4 and H19. **c)** Detailed view of mL119. Part of the protein coordinate an iron-sulfur cluster *via* four cysteine residues. The model is shown in its density. **d)** Electrostatic coloration of the mL118 protein showing the positive charge of the inner groove and the two contact points made with ES-94. **e)** Similarly mL116 by electrostatic potential revealing the largely positive surfaces both in its inner groove and exterior to interact with rRNAs.

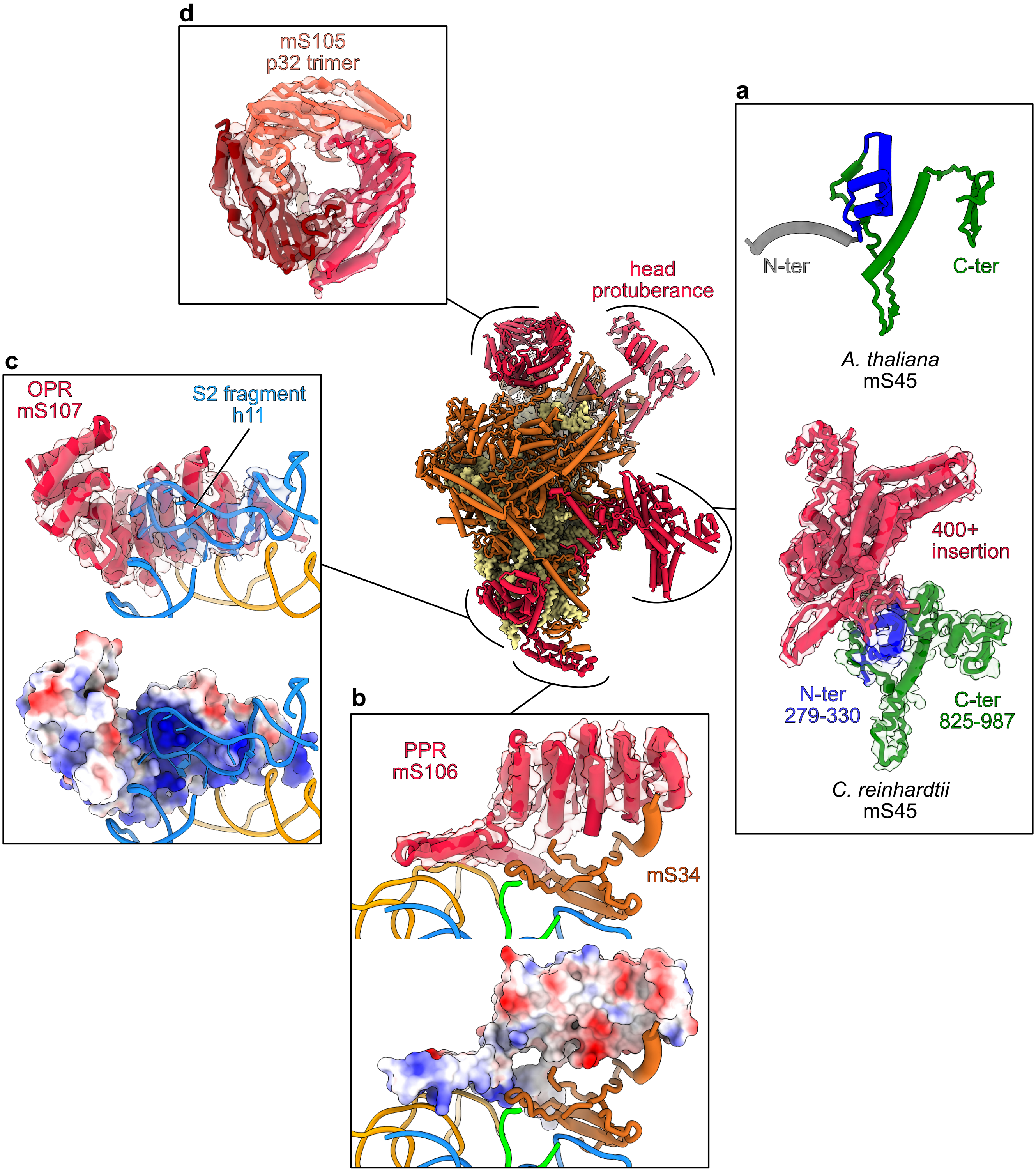

Supplementary Fig. 5. Detailed analyses and views of the specifi SSU proteins

The small subunit model is presented from the solvent view which allows to see the major structural features, highlighted in red, of the SSU. Two Y-shaped protuberances are observed on the head and the body. **a)** The body protuberance is formed by a large insertion in the conserved mS45 protein. A comparison between the Arabidopsis and Chlamydomonas mS45 proteins are shown. The head protuberance components, could not be clearly identifie and was thus entirely build as poly-alanine. However, several conserved proteins of the SSU head, notably uS3m, uS10m and mS35, have large parts that could not be modelized. These extensions could come together and form this large head protuberance. The foot extension is formed by two helical proteins, one PPR (mS106) (**b**) and one OPR (mS107) (**c**). The electrostatic potential of the proteins (blue coloration corresponds to positive patches and red to negative patches) which models were generated using AlphaFold are also presented[27]. The PPR occupies a position similar to mS27 in human[6,7] and fungi[12], but does not appear to interact with RNA, which seems consistent with the charge of the inner groove of the protein. (**c**) The OPR encapsulates the tip of helix 11. **d)** On the back of the SSU head, the homotrimeric torus-shaped domain formed by three copies of mS105 (p32) is present.

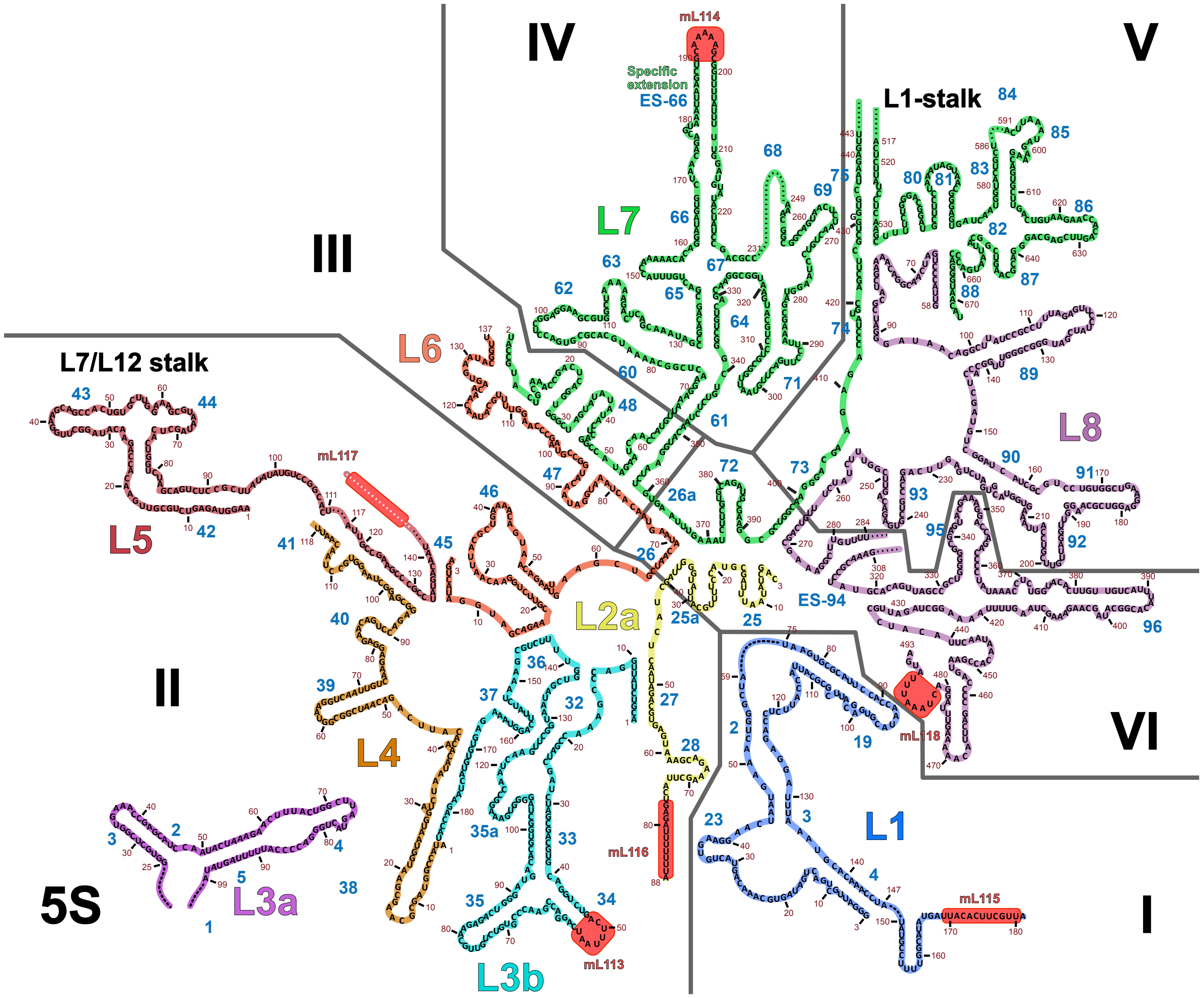

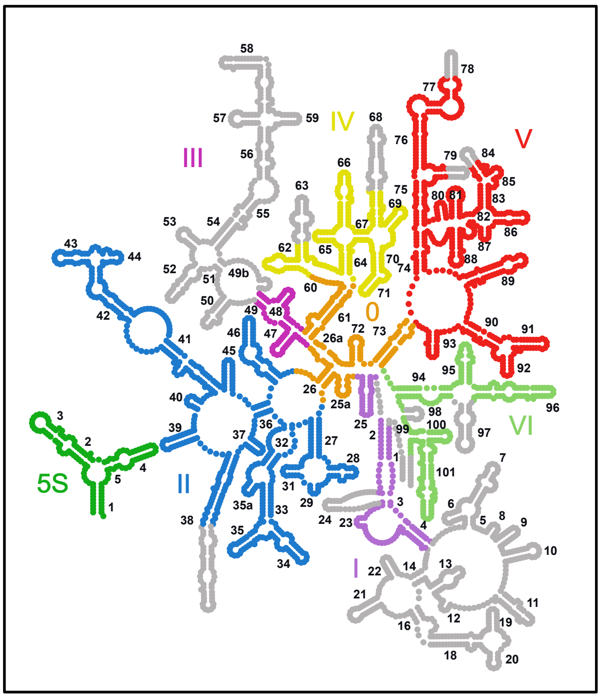

E. coli 2D diagram

Supplementary Fig. 6. 2D diagram of the *C. reinhardtii* LSU rRNAs

2D representation of the LSU rRNAs of Chlamydomonas mitoribosome. Each 9 rRNA fragments are colored differentl and match the color code of Figure 3. The rRNA expansions (ES-94 and ES-66) are indicated on the diagram. RNA portions that could not be modelled are indicated by dark dashed lines, e.g the L1 stalk could not be built due its motion. White dashed lines indicated regions that were built as polyU/A. Contact points of Chlamydomonas-specifi r-proteins are also highlighted by red boxes. Simplifie secondary structure diagram of the *E. coli* 23S rRNA is also shown in the black frame, this time colored by domain. Helices absent in Chlamydomonas mitoribosome are shown in gray, which highlight the strong reduction of domain I and III. Secondary structure templates were obtained from the RiboVision suite (http://apollo.chemistry.gatech.edu/RiboVision).

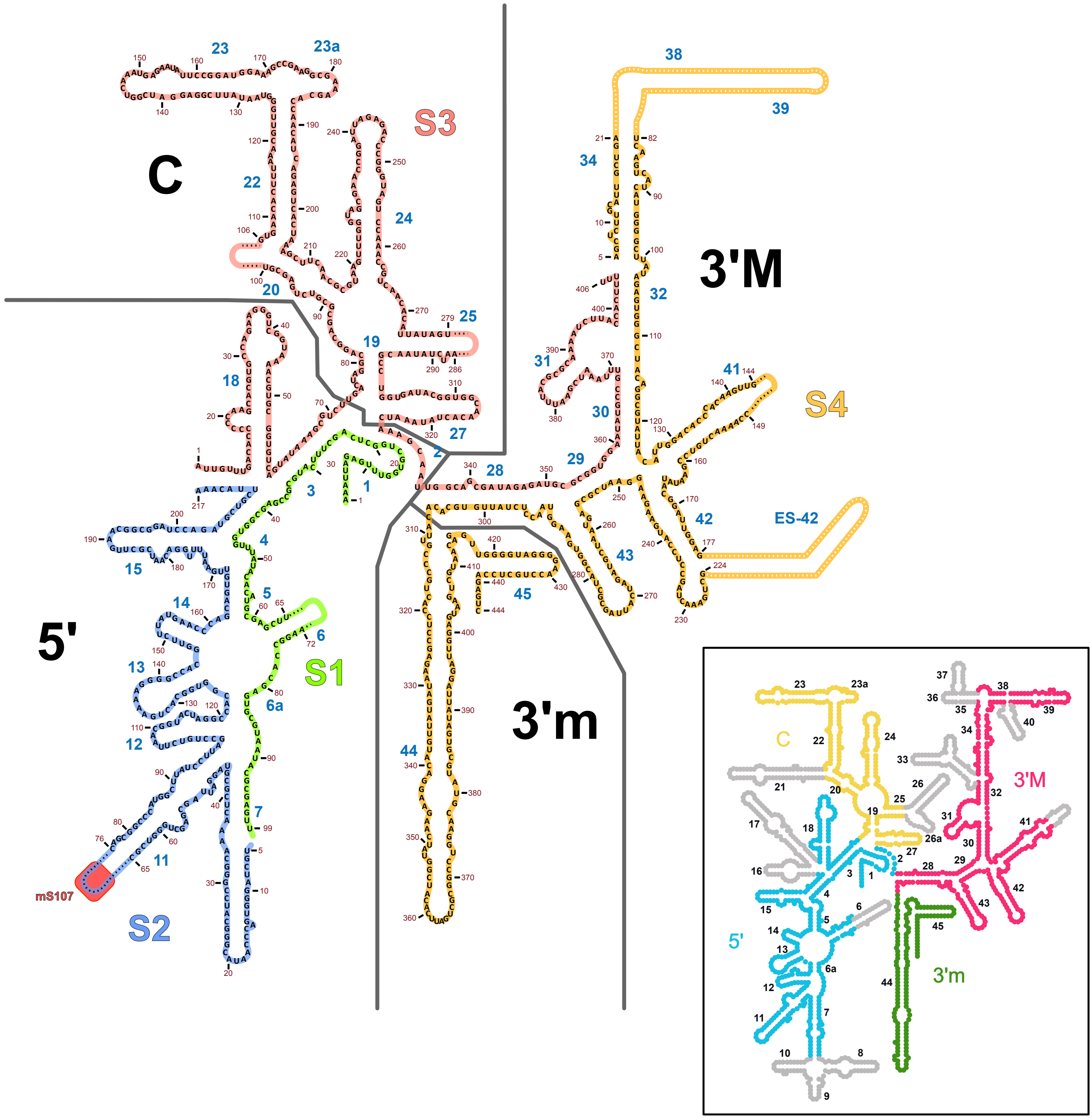

E. coli 2D diagram

Supplementary Fig. 7. 2D diagram of the *C. reinhardtii* SSU rRNAs

2D representation of the SSU rRNAs of Chlamydomonas mitoribosome. Each 4 rRNA fragments are colored differentl and match the color code of Figure 3. The rRNA expansions are indicated on the diagram. Extensions that could not be modelled are indicated by dashed lines. White dashed lines indicated regions that were built as polyU/A. Contact points of Chlamydomonas-specifi r-proteins are also highlighted by a red box. Simplifie secondary structure diagram of the *E. coli* 16S rRNA is also shown in the black frame, this time colored by domain. Helices absent in Chlamydomonas mitoribosome are shown in gray. Secondary structure templates were obtained from the RiboVision suite (http://apollo.chemistry.gatech.edu/RiboVision).

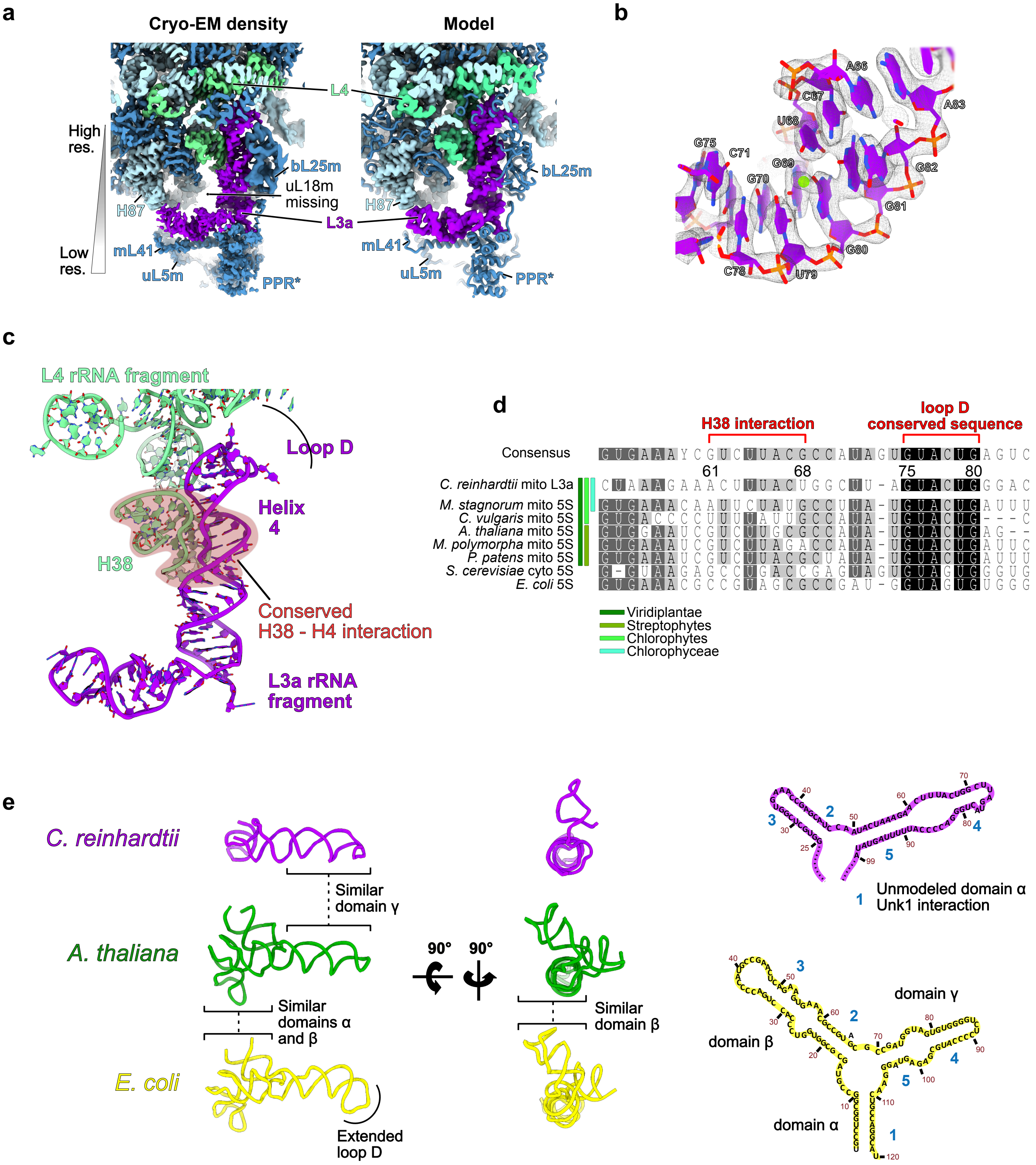

Supplementary Fig. 8. Analysis of the central protuberance, the L3a rRNA fragment is a divergent 5S
a) Comparison of the cryo-EM map of the central protuberance (CP) with the corresponding atomic model. Resolution is lower at the outer extremity of the CP due to the CP general motion. rRNAs are shown in light blue, aquamarine (L4) and purple (L3a) and proteins in blue. For the atomic model, rRNAs are shown in surface and proteins in cartoon representation. Compared to classical CP structures, the uL18 protein is missing, which is normally involved in domain α stabilization. **b)** L3a atomic model in its respective density that allowed identificatio of the rRNA fragment. **c)** The structurally conserved helix 4 area, interacting with helix 38 of the L7 fragment is highlighted and sequence alignment of the area is shown in **d**. Conservation is observed from bacteria to eukaryotes, especially at the sequence level for the pre-loop D area. The L3a fragment is particularly divergent even with member of the Chlorophytes. **e)** shows structural comparison between bacterial, mitochondrial higher plants and Chlamydomonas 5S rRNAs. Domain γ, especially helix 4 and the D loop are more similar between *A. thaliana* and *C. reinhardtii* compared to bacteria, whereas domain β is completely differ nt in *C. reinhardtii* compared to both bacteria and *A. thaliana.* Chlamydomonas domain β is angled differ ntly which allows the interaction of the fina loop of the domain with H87 which is not the case in other ribosomes. The unmodeled domain α of Chlamydomonas L3a most likely interacts with the unidentifie alpha helical protein, resembling a PPR protein, PPR*.

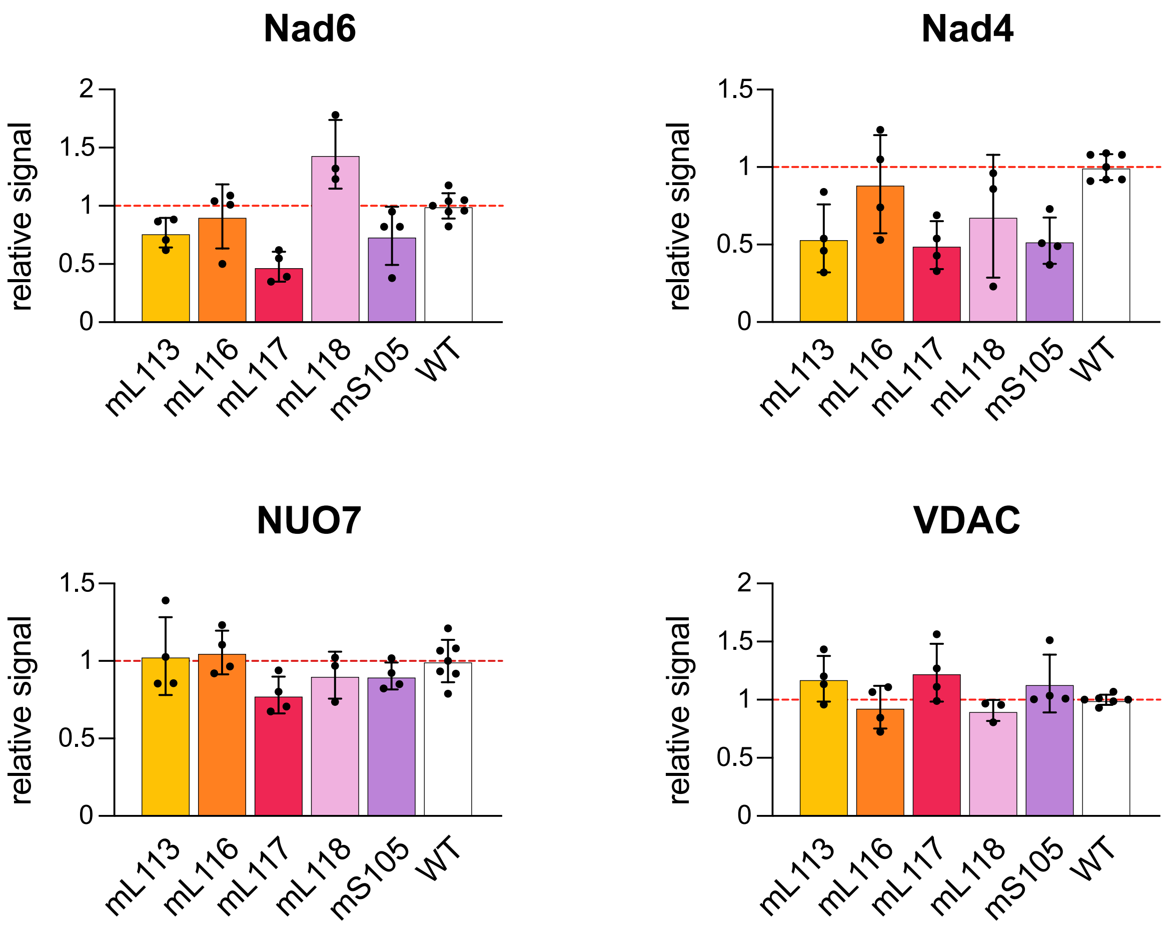

Supplementary Fig. 9. Quantification for Nad6, Nad4, NUO7, and VDAC immunoblot experiments Histograms correspond to relative signals of 3-4 technical replicates of immunoblot experiments from 2 biological replicates (see SI Table 3 and SI File). The stained blots and immunoblot signals were quantifie with ImageJ 1.52s software. The statistical difference between WT and each amiRNA strains for each signal protein as derived from the Mann-Whitney test are indicated (**p < 0.01; *p < 0.05).

Proteins of the Chlamydomonas mitoribosome

Proteins of the Large subunit

MRP	Uniprot ID	Phytozome	Chain ID	Size (aa)	Modeled residues	Comments
uL2m	A0A2K3DL58	Cre07.g348850.t1.2	a	383	69-349	
uL3m	A0A2K3E681	Cre01.g022250.t1.1	b	417	90-393	
uL4m	A0A2K3D422	Cre12.g520400.t1.2	c	427	85-424	Contacts mL115, mL116 and mL117
uL5m	A0A2K3DYN2	Cre03.g196150.t1.2	d	216	12-195	Scant density due to moving CP
uL6m	A8J503	Cre09.g415950.t1.2	e	207	32-195	Scant density but visible close de L7/L12 stalk
bL9m	A0A2K3DRF9	Cre06.g307950.t1.1	f	304	104-163	Scant density due to L1 stalk motion
uL10m	A8HVP7	Cre06.g272850.t1.2	-	235		Very scant density due to L7/L12 stalk motion in LSU reconstruction but visible in the STA data
uL11m	A8J6G9	Cre17.g722200.t1.2	-	153		Very scant density due to L7/L12 stalk motion in LSU reconstruction but visible in the STA data
uL13m	A8J810	Cre09.g405200.t1.2	i	277	6-157	
uL14m	A8IYH4	Cre12.g550650.t1.2	j	120	1-120	
uL15m	A0A2K3D8F3	Cre11.g476800.t1.1	k	337	107-316	
uL16m	A0A2K3DP67	Cre06.g278224.t1.1	l	270	135-270	
bL17m	A0A2K3DXS2	Cre03.g181350.t1.1	m	-	2-178	The protein is a mix of A0A2K3DXS2 and A8JH49
bL19m	A8I7A8	Cre07.g322500.t1.2	n	312	134-306	N-ter part stabilizes mL118
bL20m	A8HNL1	Cre01.g052350.t1.1	o	115	2-111	
bL21m	A0A2K3DDN8	Cre09.g388550.t1.2	p	370	119-341	
uL22m	A0A2K3DSG6	Cre05.g242950.t1.1	q	377	194-354	
uL23m	A8J6R9	Cre17.g727350.t1.2	r	226	30-201	
uL24m	A0A2K3DJU3	Cre07.g330450.t1.2	s	309	2-221	
bL25m	A0A2K3E0K7	Cre02.g081450.t1.2	t	366	117-361	Close to CP, scant density, contacts PPR*
bL27m	A0A2K3E880	Cre01.g053600.t1.2	u	167	-5-131	C-ter interacts with mL116. Extends in N-ter, before the first amino-acid of the reference sequence
bL28m	A0A2K3D077	Cre13.g579150.t1.2	v	153	4-123	Beta sheets stacking with the beta sheet of the N-ter of mL115
uL29m	A8HUW1	Cre13.g588310.t1.1	w	127	4-123	
uL30m	A0A2K3DSP9	Cre05.g234659.t1.1	x	214	8-213	
bL32m	A8JGW4	Cre12.g558750.t1.2	y	123	48-118	
bL33m	A8J9C3	Cre16.g653601.t1.1	z	59	5-57	
bL34m	A0A2K3CX36	Cre14.g608970.t1.1	A	280	231-280	
bL35m	A0A2K3DKX8	Cre07.g345850.t1.2	B	151	22-149	Long C-ter extension
bL36m	-	-	C	-	2-37	Present but no sequence identified
mL40	A0A2K3D9N2	Cre10.g428350.t1.2	D	172	25-118	
mL41	A8J2J1	Cre06.g291300.t1.2	E	112	25-103	
mL43	A8HW20	Cre06.g270650.t1.2	F	141	2-130	
mL46	A8IWI1	Cre03.g193550.t1.1	G	259	37-257	
mL54	A8IY84	Cre12.g546000.t1.2	-	127		Very scant density due to L7/L12 stalk motion in LSU reconstruction but visible in the STA data
mL63/57/60	A8IS96	Cre16.g684950.t1.2	I	132	10-106	C-ter extension interacts with both mL116 and mL117
mL59/64	A8HPK3	-	J	134	18-133	
mL80	A0A2K3DBX4	Cre10.g461650.t1.2	K	249	35-248	Long C-ter extension stabilize mL113
mL87	A8J535	Cre09.g417100.t1.1	L	206	59-195	N-ter additional domain contacts mL113 and domain III of reduced rRNA
mL113	A0A2K3DVD3	Cre03.g145867.t1.1	M	876	63-587	OPR (ASA2-like), contacts mL114. Contacts H34. Unmodeled C-ter part interacts with the membrane
mL114	A0A2K3DR53	Cre06.g303850.t1.1	N	455	139-424	mTERF, contacts mL113 and the specific extension ES-66
mL115	A8J070	Cre09.g392579.t1.1	O	688	75-670	OPR10, presence of Trp in several repeats. Stabilize 3' end of the L1 fragment
mL116	A0A2K3DXQ3	Cre03.g179901.t1.1	P	530	34-526	OPR (ASA2-like), contacts mL117. Stabilize 3' end of the L2a fragment
mL117	A0A2K3CXJ4	Cre14.g615450.t1.2	Q	820	130-786	OPR, contacts mL116. Stabilize the 3' end of the L5 fragment. Mostly build as polyA due to scant densi
mL118	A0A2K3DTE5	Cre04.g217904.t1.1	R	653	152-598	OPR. Stabilizes the 3' end of the L8 fragment.
mL119	A0A2K3D424	Cre12.g521050.t1.2	S	334	71-334	At the channel exit. Presence of a FeS cluster. Involved in membrane contact, probably with Oxa1
PPR*	??		Y	??	1-172	PPR-like on CP
Unk1	??		X	??	1-31	Peptide encapsulated by mL113
Unk2	??		Z	??	1-49	Next to uL25m

Proteins of the Small subunit

MRP	Uniprot ID	Phytozome	Chain ID	Size (aa)	Modeled residues	Comments
bS1m	A8IB56	Cre10.g432200.t1.2	a	253	40-253	Clearly visible
uS2m	A0A2K3CXL0	Cre14.g614950.t1.2	b	530	118-440	
uS3m	A0A2K3D7R0	Cre11.g467702.t1.1	c	490	126-260	Unmodelled residues are most likely part of the unknown proteins of the head
uS4m	A0A2K3E198	Cre02.g091450.t1.1	d	415	257-385	
uS5m	A0A2K3DFB5	Cre09.g407100.t1.1	e	368	175-350	
bS6m	A8JGX6	Cre12.g558000.t1.1	f	122	2-104	
uS7m	A0A2K3CXT8	Cre14.g618700.t1.1	g	281	144-281	
uS8m	A0A2K3CNM3	Cre17.g696550.t1.2	h	412	10-371	
uS9m	A0A2K3E2Q3	Cre02.g106750.t1.2	i	446	284-437	
uS10m	A0A2K3CN25	Cre24.g755297.t1.1	j	580	112-290	Unmodelled residues are most likely part of the unknown proteins of the head
uS11m	A0A2K3DQ48	Cre06.g288400.t1.1	k	233	121-219	Very scant density of the platform area
uS12m	A0A2K3E3V0	Cre02.g144400.t1.1	l	128	13-122	
uS13m	A8J3J1	Cre16.g674350.t1.2	m	124	18-116	
uS14m	A8J6P9	Cre17.g726300.t1.1	n	112	15-111	
uS15m	A0A2K3CQY8	Cre17.g731250.t1.2	o	300	107-287	
bS16m	A0A2K3D9S4	Cre10.g429550.t1.2	p	88	1-79	
uS17m	A0A2K3DKB4	Cre07.g337800.t1.2	q	220	5-184	
bS18m	A0A2K3DDW6	Cre09.g388282.t1.1	r	356	238-293	
uS19m	A0A2K3DHN0	Cre08.g375250.t1.1	s	116	4-94	
bTHXm	A8J1A6	Cre09.g391208.t1.1	u	136	66-89	
mS23	A8IW03	Cre12.g530500.t1.2	v	190	9-136	
mS26	A8J4B1	Cre08.g361500.t1.2	w	193	34-188	
mS29	A0A2K3DXG4	Cre03.g176700.t1.1	x	485	63-479	
mS33	A8JI15	Cre17.g747697.t1.1	y	209	125-185	
mS34	A8J254	Cre06.g283350.t1.1	z	133	22-115	
mS35	A0A2K3CST5	Cre16.g650700.t1.1	B	883	818-870	Unmodelled residues are most likely part of the unknown proteins of the head
mS45	A0A2K3DM91	Cre06.g254600.t1.1	C	999	280-600/853-980	Large insertion and N-terminal could not be modeled and likely form most of the body protuberance
mS45-in	A0A2K3DM91	Cre06.g254600.t1.1	D	-		Probable insertion and N-terminal part of mS45 modeled as a separate block
mS31/46	-	-	K	-	1-186	Present but no sequence identified
			L			
mS105	A0A2K3DAY3	Cre10.g447100.t1.2	M	253	69-251	p32 trimer
			N			
mS106	A0A2K3DZV7	Cre03.g211409.t1.1	O	516	271-513	PPR at the foot. Similar to mS27 (human and yeast) or mS90 in tetrahymena. AlphaFold modelization
mS107	A0A2K3CRX4	Cre17.g743597.t1.1	P	416	1-413	OPR SSU, contact h11. AlphaFold modelization
Unk1	??		U	??	1-22	Unknown body protein
Unk2	??		V	??	1-218	Unknown helical protein(s) bundle on the body
Unk3	??		W	??	1-54	Unknown peptide in the body protuberance
Unk4	??		X	??	1-91	Unknown helical bundle on the head
Unk5	??		Y	??	1-92	Unknown peptide linking head and body and contacting mS105
Unk6	??		Z	??	1-695	Unknown head protuberance, most likely formed by extensions of conserved proteins

Supplementary Table 1. List of proteins identifie as constituent *C. reinhardtii* mitoribosome, for the LSU and SSU

List of the r-proteins constituting the *C. reinhardtii* mitoribosome, the table is divided between LSU and SSU r-proteins. The proteins are colored by conservation with the bacterial ribosome (blue) other mitochondrial ribosomes (yellow) or specifi to *C. reinhardtii* mitoribosome (red). Due to the L7/L12 stalk motion, proteins uL10m, uL11m and mL54 were not visualized and are presented only in the illustration figures not the deposited fina model. Similarly, bL12m was observed in the subtomogram averaging reconstruction but is not present in the fina model. Due to the overall lower resolution of the SSU compared to the LSU, the totality of visible extensions and insertion were modelled as polyA. bS21m, mS37 and mS38 are most likely present but could not be observed in our reconstruction. Full list of proteins identifie is provided in Supplementary information.

	#1 LSU (EMD-13480)	#2 SSU body (EMD-13477)	#3 SSU head (EMD-13481)
	(PDB 7PKT)	(PDB 7PKQ)	
**Data collection and processing**			
Magnification	45,000X	36,000X	
Voltage (kV)	200	200	
Electron exposure (e–/Å^2)	45	45	
Defocus range (μm)	-0.5 to -2.5	-0.5 to -2.5	
Pixel size (Å)	0.9	1.13	
Symmetry imposed	C1	C1	
Initial particle images (no.)	346,994	445,469	
Final particle images (no.)	101,291	40,131	
Map resolution (Å)<br>FSC threshold	2.93<br>0.143	4.19<br>0.143	4.47<br>0.143
Map resolution range (Å)	2.7 - 7	3.8 - 10	
**Refinement**			
Initial model used (PDB code)	5KCR, 6GAW, 6XYW, 5MRC		
Map sharpening *B* factor (Å^2)	-50.6	-169.5	-174.3
Model composition<br>Non-hydrogen atoms<br>Protein residues<br>Nucleotide residues<br>Ligands	57 chains<br>103,142<br>8300<br>1901<br>Zn:1 Mg:69 FES:1	44 chains<br>70,328<br>6690<br>1114<br>-	
FSC (model) = 0.143 (Å)<br>= 0.5 (Å)<br>CC	3.00<br>3.18<br>0.75	4.12<br>4.35<br>0.73	4.38<br>5.01<br>0.75
B factors (Å^2) (mean)<br>Protein<br>Nucleotide<br>Ligand	 27.87<br>36.85<br>19.13	 101.52<br>143.00<br>-	
R.m.s. deviations<br>Bond lengths (Å)<br>Bond angles (°)	 0.010<br>1.228	 0.007<br>1.223	
Validation<br>MolProbity score<br>Clashscore<br>Poor rotamers (%)	 1.72<br>5.50<br>0.26	 1.88<br>8.12<br>0.21	
Ramachandran plot<br>Favored (%)<br>Allowed (%)<br>Disallowed (%)	 93.56<br>6.19<br>0.25	 93.27<br>6.57<br>0.15	
RNA validation<br>Sugar pucker outliers (%)<br>RNA backbone	 1%<br>0.46	 1%<br>0.38	

Supplementary Table 2. Cryo-EM data collection, refinement and validation statistics

Gene	Genome	Gene ID	Experiment	Forward primer (5' -> 3')	Reverse primer (5' -> 3')
CYN19-3	Nucleus	*Cre13.g588100.t1.2*	qPCR	CAAGACTGCGGAGAACTTCC	CACATGAAGGAGGGGATGAC
MAA7	Nucleus	*Cre03.g161400.t1.2*	qPCR	GTTCGGCGGCAAGTACGT	CGCAATCGCCTCGTTGTAGT
mL113	Nucleus	*Cre03.g145867.t1.1*	qPCR	GACAGCTATCTCTGTCTTTGTTAGCTC	ACTCCTCTCCCGACAGCAC
mL113	Nucleus	*Cre03.g145867.t1.1*	amiRNA	ctagtTCGCATCAGTATAACTAGATAtctcgctgatcggcaccatgggggtggtggtgatcagcgctaTATCAAGTTATACTGATGCGAg	ctagcTCGCATCAGTATAACTTGATAtagcgctgatcaccaccacccccatggtgccgatcagcgagaTATCTAGTTATACTGATGCGAa
mL116	Nucleus	*Cre03.g179901.t1.1*	qPCR	CTCAGACCGGCCAACATC	TGAACTCTCAGCGCGTAGG
mL116	Nucleus	*Cre03.g179901.t1.1*	amiRNA	ctagtAAGATCAACACCTCTGTAAGAtctcgctgatcggcaccatgggggtggtggtgatcagcgctaTCTTTCAGAGGTGTTGATCTTg	ctagcAAGATCAACACCTCTGAAAGAtagcgctgatcaccaccacccccatggtgccgatcagcgagaTCTTACAGAGGTGTTGATCTTa
mL117	Nucleus	*Cre14.g615450.t1.2*	qPCR	CAGCTGCAGCAACACCAG	CGACACGTTGGACTGGAAC
mL117	Nucleus	*Cre14.g615450.t1.2*	amiRNA	ctagtCAGGCTGAACAAACTTGAACAtctcgctgatcggcaccatgggggtggtggtgatcagcgctaTGTTTGAGTTTGTTCAGCCTGg	ctagcCAGGCTGAACAAACTCAAACAtagcgctgatcaccaccacccccatggtgccgatcagcgagaTGTTCAAGTTTGTTCAGCCTGa
mL118	Nucleus	*Cre04.g217904.t1.1*	qPCR	ATCCCCAGCGTCCAATCT	TTCGGAGCTGTTGGTGGT
mL118	Nucleus	*Cre04.g217904.t1.1*	amiRNA	ctagtCCGAAAGGAGGTCTGTGATTAtctcgctgatcggcaccatgggggtggtggtgatcagcgctaTAATGACAGACCTCCTTTCGGg	ctagcCCGAAAGGAGGTCTGTCATTAtagcgctgatcaccaccacccccatggtgccgatcagcgagaTAATCACAGACCTCCTTTCGGa
mS105	Nucleus	*Cre10.g447100.t1.2*	qPCR	TGATGAGCTGGATGACAACCT	GAGGAAGCGCAGGTACTCC
mS105	Nucleus	*Cre10.g447100.t1.2*	amiRNA	ctagtGTGATCGCGTATTGCTCTTTAtctcgctgatcggcaccatgggggtggtggtgatcagcgctaTAAACAGCAATACGCGATCACg	ctagcGTGATCGCGTATTGCTGTTTAtagcgctgatcaccaccacccccatggtgccgatcagcgagaTAAAGAGCAATACGCGATCACa
rrnL1	Mitochondria		qPCR	AGGGATTGCTGACTGATAGTGC	ATGCGCGAATGCTCACGTATTG
rrnL2a	Mitochondria		qPCR	GCTTTCTGCTTAACTCACG	GCGTACCTTTTGCATTATGG
rrnL2b	Mitochondria		qPCR	GCGTGCAACAACACACTAAAGGG	TGTGAGAGTGTGGGTTGAACCAG
rrnL3a	Mitochondria		qPCR	ACTCAATTTCGGTGCTCGGTGA	GGGTCCCAGTACTAAGCCAGTAA
rrnL3b	Mitochondria		qPCR	AACCGATCGATCTAGCGACGTG	TCTTGCAACAGACACGGGTTCG
rrnL4	Mitochondria		qPCR	ATACCGGTAGCGCAAGCGAATG	TGACCTTACCGCCGATTGTCTG
rrnL5	Mitochondria		qPCR	AAGGTAGAGTCTGCGTTGAACACC	TTCCAAGCACAGTGGCTGCTTC
rrnL6	Mitochondria		qPCR	ACGTTCTGGAACAATTACGCTGA	GGGCTTACGGCCATGTTATCCA
rrnL7	Mitochondria		qPCR	ACGTTTAATTGGCGTCCTTG	AGCTGCATAGGGTCTCTTCG
rrnL8	Mitochondria		qPCR	CGCCTATGCACAGTTAGCC	TGACAACAAGTGTACCAGAGGTTT
rrnS1	Mitochondria		qPCR	GTTTGGTGCTGGCTCAGCTTTC	ATTACGCACTCGTGCGCTTAATC
rrnS3	Mitochondria		qPCR	TTCAAATCTATAACGCCTGGTG	GCTGCCAATTGCTTTGATTT
rrnS4	Mitochondria		qPCR	ACGGTCGAGCAAGTCATCATGG	TGGTATCCAACTTGTGGGTGTCC
rtl	Mitochondria		qPCR	TACGCCGACAGTACATCCAG	TACCAAAACCAGGACGGAAG
dT			RT		TTTTTTTTTTTTTTTTTTTTTTTT

Supplementary Table 3. List of oligonucleotides used in this study
