# Supplementary material for: How to build a ribosome from RNA fragments in Chlamydomonas mitochondria
Source: Nat Commun. 2021 Dec 9;12:7176. doi: 10.1038/s41467-021-27200-z (PMC8660880; doi:10.1038/s41467-021-27200-z)
Supplement: Supplementary file 3 — Description of Additional Supplementary Files [file 41467_2021_27200_MOESM3_ESM.pdf]

Description of Additional Supplementary Files

File Name: Supplementary Movie 1
Description: Overall presentation of the C. reinhardtii mitoribosome

File Name: Supplementary Movie 2
Description: In situ visualization of C. reinhardtii mitoribosomes
