# Supplementary material for: How to build a ribosome from RNA fragments in Chlamydomonas mitochondria
Source: Nat Commun. 2021 Dec 9;12:7176. doi: 10.1038/s41467-021-27200-z (PMC8660880; doi:10.1038/s41467-021-27200-z)
Supplement: Supplementary file 6 — Reporting Summary [file 41467_2021_27200_MOESM6_ESM.pdf]

nature portfolio

Corresponding author(s): Philippe Giégé
Laurence Drouard
Yaser Hashem

Last updated by author(s): Nov 4, 2021

Reporting Summary

Nature Portfolio wishes to improve the reproducibility of the work that we publish. This form provides structure for consistency and transparency in reporting. For further information on Nature Portfolio policies, see our Editorial Policies and the Editorial Policy Checklist.

Statistics

For all statistical analyses, confirm that the following items are present in the figure legend, table legend, main text, or Methods section.

n/a	Confirmed	
☐	☒	The exact sample size (*n*) for each experimental group/condition, given as a discrete number and unit of measurement
☐	☒	A statement on whether measurements were taken from distinct samples or whether the same sample was measured repeatedly
☐	☒	The statistical test(s) used AND whether they are one- or two-sided<br>*Only common tests should be described solely by name; describe more complex techniques in the Methods section.*
☒	☐	A description of all covariates tested
☐	☒	A description of any assumptions or corrections, such as tests of normality and adjustment for multiple comparisons
☐	☒	A full description of the statistical parameters including central tendency (e.g. means) or other basic estimates (e.g. regression coefficient) AND variation (e.g. standard deviation) or associated estimates of uncertainty (e.g. confidence intervals)
☐	☒	For null hypothesis testing, the test statistic (e.g. *F*, *t*, *r*) with confidence intervals, effect sizes, degrees of freedom and *P* value noted<br>*Give P values as exact values whenever suitable.*
☒	☐	For Bayesian analysis, information on the choice of priors and Markov chain Monte Carlo settings
☒	☐	For hierarchical and complex designs, identification of the appropriate level for tests and full reporting of outcomes
☒	☐	Estimates of effect sizes (e.g. Cohen's *d*, Pearson's *r*), indicating how they were calculated

Our web collection on statistics for biologists contains articles on many of the points above.

Software and code

Policy information about availability of computer code

Data collection	The single particle data collection was performed on a Talos Arctica instrument (Thermofisher Company) at 200 kV using the SerialEM software for automated data acquisition. Data were collected at a nominal underfocus of -0.5 to -2.5 µm at a magnification of 36,000 X yielding a pixel size of 1.13 Å for the SSU and 45,000 X yielding a pixel size of 0.9 Å for the LSU. Micrographs were recorded as movie stack on a K2 direct electron detector (GATAN Company), each movie stack were fractionated into 65 frames for a total exposure of 6.5 sec corresponding to an electron dose of 45 ē/Å2.<br>Cellular tomograms were acquired on a 300 kV Titan Krios microscope (FEI), equipped with a Gatan post-column energy filter (968 Quantum) and a direct detector camera (K2 summit, Gatan) operated in movie mode at 12 frames per second. Tilt series were recorded using SerialEM software with 2° tilt increments from −60° to +60° (in two halves separated at either 0° or −20°), an object pixel size of 3.42 Å, a defocus of −4 to −5.5 µm, and a total accumulated dose of <100 e−/Å.<br>For protein steady-state level analyses, staining and immunoblot signals were acquired with the FUSION FXcdevice (Vilbert Loumart) and quantified with ImageJ 1.52s software.
Data analysis	MotionCorr2, Gctf v1.06, RELION 3.0, cryoSPARC v3.0, ResMap 1.95, Swiss-model (latest online version), Coot 0.9, Phenix 1.18, Chimera 1.13.1, ChimeraX 1.1, EMAN2, TOM ToolBox

For manuscripts utilizing custom algorithms or software that are central to the research but not yet described in published literature, software must be made available to editors and reviewers. We strongly encourage code deposition in a community repository (e.g. GitHub). See the Nature Portfolio guidelines for submitting code & software for further information.

Data

Policy information about availability of data

All manuscripts must include a data availability statement. This statement should provide the following information, where applicable:

- Accession codes, unique identifiers, or web links for publicly available datasets
- A description of any restrictions on data availability
- For clinical datasets or third party data, please ensure that the statement adheres to our policy

The cryo-EM maps of C. reinhardtii mitoribosome have been deposited at the Electron Microscopy Data Bank (EMDB): EMD-13480 for the LSU, EMD-13481 for the head of the SSU, EMD-13477 for the body of the SSU and EMD-13578 for the subtomogram average of the whole ribosome. The corresponding atomic models have been deposited in the Protein Data Bank (PDB) under the accession 7PKT for the LSU and 7PKQ for the SSU. Mass spectrometric data have been deposited to the ProteomeXchange Consortium via the PRIDE partner repository with the dataset identifier PXD024708 and 10.6019/PXD024708. RNAseq data were deposited in the NCBI Gene Expression Omnibus under accession number GSE171125.

Field-specific reporting

Please select the one below that is the best fit for your research. If you are not sure, read the appropriate sections before making your selection.

- [x] Life sciences
- [ ] Behavioural & social sciences
- [ ] Ecological, evolutionary & environmental sciences

For a reference copy of the document with all sections, see nature.com/documents/nr-reporting-summary-flat.pdf

Life sciences study design

All studies must disclose on these points even when the disclosure is negative.

Sample size	For Cryo-EM, one biological sample was analyzed resulting from one of the two purifications performed. From that purification resulted two reconstructions, one for the large and small subunit.
Data exclusions	No data were excluded.
Replication	Two purification experiments were repeated in the same conditions and always successfully yielded the same complexes when analyzed by cryo-EM and mass spectrometry. For rRNA accumulation analyses by qRT-PCR, the averages were obtained from three biological replicates, each analyzed in three technical replicates. For Chlamydomonas phenotype analyses, experiments were repeated at least two times. For protein steady-state level analyses, the averages were obtained from two biological replicates, each analyzed in three to four technical replicates.
Randomization	The randomization was not necessary since one biological sample was analyzed to our study.
Blinding	We had no prior knowledge of the complex of interest, and blinding was unnecessary to our study.

Reporting for specific materials, systems and methods

We require information from authors about some types of materials, experimental systems and methods used in many studies. Here, indicate whether each material, system or method listed is relevant to your study. If you are not sure if a list item applies to your research, read the appropriate section before selecting a response.

Materials & experimental systems

n/a	Involved in the study	
[ ]	[x]	Antibodies
[x]	[ ]	Eukaryotic cell lines
[x]	[ ]	Palaeontology and archaeology
[x]	[ ]	Animals and other organisms
[x]	[ ]	Human research participants
[x]	[ ]	Clinical data
[x]	[ ]	Dual use research of concern

Methods

n/a	Involved in the study	
[x]	[ ]	ChIP-seq
[x]	[ ]	Flow cytometry
[x]	[ ]	MRI-based neuroimaging

Antibodies

Antibodies used	Specific primary antibodies used in immunoblotting were provided by Pr. Claire Remacle except the VDACI primary antibody provided by Dr. Thalia Salinas-Giegé. They were detected using goat anti-rabiit IgG, horseradish peroxidase conjugate (Invitrogen#G21234) and chemiluminescence (Clarity Western ECL Substrate, Bio-Rad) with the FUSION FX device (Vilbert Lourmat). The primary antibodies correspond to rabbit sera obtained against Chlamydomonas reinhardtii mitochondrial-encoded sub-units complex I, Nad4 (1:1000)

	and Nad6 (1:100), nuclear-encoded sub-unit complex I, NUO7 (1:2000), and the nuclear-encoded mitochondrial protein VDACI (1:25000).
Validation	Primary antibodies Nad4, Nad6 and NUO7 were validated by Pr. Claire Remacle and VDACI primary antibody was validated by Dr. Thalia Salinas-Giégé. Their reactivity was confirmed on Chlamydomonas reinhardtii mitochondrial extract. The expected/apparent molecular weight are as follow: 49kDa/50-55kDa for Nad4, 18kDa/18kDa for Nad6, 49kDa/38kDa for NUO7 and 28kDa/28kDa for VDACI.
